# Comparative phylogenomics of ESBL-, AmpC- and carbapenemase-producing *Klebsiella pneumoniae* originating from companion animals and humans

**DOI:** 10.1093/jac/dkac041

**Published:** 2022-02-28

**Authors:** Raquel Garcia-Fierro, Antoine Drapeau, Melody Dazas, Estelle Saras, Carla Rodrigues, Sylvain Brisse, Jean-Yves Madec, Marisa Haenni

**Affiliations:** 1 Unité Antibiorésistance et Virulence Bactériennes, Université Claude Bernard Lyon 1 - ANSES, Lyon, France; 2 Institut Pasteur, Université de Paris, Biodiversity and Epidemiology of Bacterial Pathogens, Paris, France

## Abstract

**Background:**

WHO considers ESBL- and carbapenemase-producing *Klebsiella pneumoniae* a major global concern. In animals, ESBL- and carbapenemase-producing *K. pneumoniae* of human-related ST11, ST15 and ST307 have been reported, but not in the context of large WGS-based One Health investigations.

**Objectives:**

To perform comparative phylogenomics on a large collection of multidrug-resistant (MDR) *K. pneumoniae* recovered from diseased companion animals and humans.

**Methods:**

MDR *K. pneumoniae* (*n* = 105) recovered from companion animals in France during 2010–18 were phenotypically characterized. All isolates were whole-genome sequenced using the NovaSeq technology and phylogenomic analysis across animal and human *K. pneumoniae* was performed using appropriate pipelines.

**Results:**

*bla*_CTX-M-15_, *bla*_DHA-1_ and *bla*_OXA-48_ were strongly associated with IncFIIk, IncR and IncL plasmids, respectively. When compared with human *K. pneumoniae* genomes, four groups of closely related French human and animal isolates belonging to ST11, ST15 and ST307 were detected, suggesting the circulation of clones between the human and animal sectors at country level. A large cluster of 31 ST11-KL105 animal isolates from France and Switzerland suggested it corresponds to a sub-lineage that is particularly well-adapted to the animal host.

**Conclusions:**

This study demonstrates the spread of *bla*_CTX-M-15_-carrying ST15 and ST307, and *bla*_DHA-1_-carrying ST11 *K. pneumoniae* clones in animal populations. ST11 was the main vector of *bla*_OXA-48_/IncL, despite the absence of carbapenem use in French animals. Comparative phylogenomics suggests cross-transmission of *K. pneumoniae* sub-lineages more prone than others to colonize/infect the animal host. Our data also evidenced the emergence of convergent hypervirulent and MDR *K. pneumoniae* in animals.

## Introduction

*Klebsiella pneumoniae* is ubiquitous in nature and an inhabitant of the gastrointestinal tract of healthy humans and animals.^1,2^ It is also a common opportunistic hospital-acquired pathogen causing several types of mild to severe infections.^3–5^ In recent decades, *K. pneumoniae* has emerged as a major driver of antimicrobial resistance (AMR) through the production of ESBLs and carbapenemases, and was recognized by the WHO as one of the global priority pathogens in critical need of next-generation antibiotics and new control strategies.^6^

Human-associated clones have been reported in pets, e.g. ST15/*bla*_CTX-M-15_*K. pneumoniae* has been detected in hospital-acquired infections in cats and dogs in France, Italy, Germany, Portugal, Japan and Taiwan,^7–13^ ST11/*bla*_DHA-1_ has been found in companion animals in Spain, Switzerland and Germany,^9,14,15^and ST307 has been detected in companion animals in Portugal, Japan, South Korea and Brazil.^1,16–19^ Finally, most *bla*_OXA-48_ genes reported in companion animals in Germany and Spain were spread by ST11 and ST15 isolates, mirroring the human epidemiology.^9,20,21^ The fact that ESBL-, AmpC- and/or carbapenemase-producing *K. pneumoniae* found in humans and animals mostly belonged to the same major STs (11, 15 and 307)^4^ and isolates of *K. pneumoniae* producing the carbapenemase OXA-48 have been detected in companion animals despite prohibited or strictly regulated veterinary use of carbapenems,^9^ reinforce the argument supporting human-to-animal transmissions and vice versa. Also, it has been proven that humans and animals from the same household can share similar *K. pneumoniae* lineages.^2^

On the other hand, there are still only a very few and fragmented WGS-based studies on this bacterial species in the animal sector (in contrast to *Escherichia coli*). Consequently, advanced phylogenetic information on MDR *K. pneumoniae* in veterinary medicine regarding human *K. pneumoniae* datasets remains highly limited. This study aimed to fill this gap by providing a new comprehensive understanding of the phylogenomic relatedness of the major ESBL/AmpC/carbapenemase-producing *K. pneumoniae* lineages/sub-lineages recognized in animals and humans.

## Materials and methods

### Bacterial isolates

Non-duplicate cefoxitin- and/or ceftiofur-resistant *K. pneumoniae* isolates (*n* = 105) were recovered from diseased companion animals between 2010 and 2018 in France through the national surveillance network of AMR in animal pathogens (Resapath; http://www.resapath.anses.fr) (Table S1, available as Supplementary data at *JAC* Online). The clinical specimens were isolated from dogs (*n* = 63), cats (*n* = 28), horses (*n* = 10), cattle (*n* = 3) and birds (*n* = 1). Identification was performed using MALDI-TOF VITEK MS Version 3.0 (bioMérieux, Marcy L’Etoile, France).

### Antimicrobial susceptibility testing

Antimicrobial susceptibility tests were performed by the disc diffusion method, using commercially available discs (Mast Diagnostic, Amiens, France) according to the guidelines of the Antibiogram Committee of the French Society for Microbiology (https://www.sfm-microbiologie.org/). Susceptibility to 32 antibiotics of veterinary and human interest was tested (Table S2). ESBL production was confirmed by double-disc synergy test and the AmpC phenotype was suspected in case of cefoxitin resistance. *E. coli* ATCC 25922 was used as the quality control strain.

### Plasmid analysis and location of resistance genes

Plasmid sizes were estimated by S1-PFGE gels.^22^ To localize β-lactamase genes on specific plasmids, isolates were further analysed by Southern blot using adequate DIG-labelled probes (Roche Applied Science, Meylan, France) according to the manufacturer’s protocol. Probes for *bla*_CTX-M-type_, *bla*_SHV-2_, *bla*_DHA-1_, *bla*_OXA-48_ and *bla*_CMY-2_ were used, as well as probes corresponding to the various adequate replicon types found. The chromosomal location of β-lactamase genes was assessed by hybridization of I-*Ceu*I digested DNA with *bla*_CTX-M-15_ and a 16S rDNA probe.

### Genome sequencing

Bacterial genomic DNA was extracted using the NucleoSpin® Microbial DNA Kit (Machery-Nagel, Hoerdt, France) according to the manufacturer’s instructions. DNA concentration and purity were respectively determined using the Qubit R 3.0 Fluorometer (Thermo Fisher Scientific, Illkirch, France) and the NanoDrop 1000 (Ozyme, Saint-Cyr-l’Ecole, France). Library preparation was performed using the Nextera XT DNA library preparation kit and 2 × 150 paired-end sequencing was performed using the NovaSeq 6000 Illumina technology (Illumina, San Diego, USA). Illumina adapter sequences were removed and reads were quality trimmed using trimmomatic version 0.39.^23^*De novo* assemblies were generated with Shovill version 1.0.0 (https://github.com/tseemann/shovill), and the quality of assemblies was assessed using QUAST v4.5.1.^24^

### Genome sequencing analysis

STs and core genome MLST (cgMLST) were assigned using the online BIGSdb *K. pneumoniae* database available on the Institut Pasteur MLST and whole-genome MLST website (https://bigsdb.pasteur.fr/klebsiella/).^4^ New alleles and STs were submitted to the MLST database. Kleborate and BIGSdb analytical tools were used to detect capsular loci (KL types), *K. pneumoniae* virulence determinants and heavy metal tolerance genes. AMR genes were assessed using Kleborate (https://github.com/katholt/Kleborate) and ResFinder, whereas plasmid replicons were obtained using PlasmidFinder (http://www.genomicepidemiology.org). In addition to the Southern blot hybridizations, plasmid location of AMR genes was evaluated for all isolates based on the co-localization of resistance and plasmid incompatibility group genes on the same contig.

### Clonal relationships

Two datasets were defined, one including French animal isolates recovered in this study, and a second comprising French animal isolates belonging to ST11 and its single-locus variant (SLV) ST4850, ST15 and ST307 together with the whole genome sequences from humans belonging to the same STs and retrieved from the European Survey of Carbapenemase-Producing Enterobacteriaceae (EuSCAPE) study,^25^ as well as additional animal isolates from different countries also belonging to these STs.^26^

All assemblies were annotated with Prokka version 1.12 using default settings,^27^ and for both datasets, a pangenome was determined and core gene alignments were generated using Roary v. 3.11.0 with a protein BLAST identity of 80% and a core definition of 90%.^28^ Subsequently, recombination was removed with Gubbins v2.3.4 and a maximum likelihood tree was constructed from the recombination-purged core gene alignment using RAxML v.8.2.8 and 100 bootstrap repeats. Snp-dists version 0.7.0 (https://github.com/tseemann/snp-dists) was used to extract the number of SNPs between all isolates from the recombination-free core genome alignments. The resulting trees for both analysis were visualized using iTol v.5.5.1 (http://itol.embl.de/itol.cgi). For both datasets a minimum-spanning tree (MST) based on cgMLST was also constructed and visualized using the plugin GrapeTree integrated in BIGSdb.^29^ For human isolates, we focused on the 326 ST11, ST15 and ST307 genomes present among the 1717 human *K. pneumoniae* genomes investigated in the wide-scale EuSCAPE study, which included carbapenem non-susceptible and susceptible isolates.

### Statistics

To test the possible correlation between an animal host and the occurrence of a gene, we applied the chi^2^ test (or the Fisher test when the numbers were small).

### Data access

The whole genome shotgun project was deposited in DDBJ/EMBL/GenBank under the BioProject accession number PRJNA675776.

### Ethics

No ethics approval was needed since all samples were taken during a veterinary visit and for diagnostic purposes.

## Results

### Antimicrobial susceptibility data and resistance determinants

All identified isolates belonged to the *K. pneumoniae* species complex, with *K. pneumoniae* (Kp1) being dominant (98%; 103/105). The 105 *K. pneumoniae* isolates were resistant to cefoxitin (*n* = 13), ceftiofur (*n* = 50) or to both (*n* = 42). Based on WGS data, 52 isolates presented an ESBL gene, 34 an AmpC gene and 7 co-harboured both genes. ESBL producers predominantly harboured *bla*_CTX-M-15_ (80.8%, *n* = 42/52) (Table 1), while *bla*_DHA-1_ was by far the most frequent AmpC gene (91.2%, *n* = 31/34). The *bla*_OXA-48_ gene was detected in 12 isolates (11.4%, *n* = 12/105), mostly in combination with *bla*_DHA-1_ (*n* = 9). No statistical correlation between any animal species and specific resistance genes was detected among the isolates studied (*P >* 0.05 for all combinations). Antimicrobial susceptibility testing revealed that all *K. pneumoniae* were MDR, with proportions of resistances to sulfonamides, quinolones and fluoroquinolones exceeding 80%. Susceptibility was largely retained for amikacin and netilmicin with resistance rates of 1.9% (*n* = 2/105) and 5.7% (*n* = 6/105), respectively (Table 2 and Table S2). The *armA* gene was detected in one isolate retrieved from a horse and harbouring *bla*_SHV-12_ and *bla*_DHA-1_. Colistin resistance genes were found in two isolates, one displaying a mutated *mgrB* gene (MIC of 16 mg/L) and one presenting the inducible *mcr-9* gene (MIC of 0.064 mg/L).

**Table 1.** Distribution of β-lactam resistance genes among *K. pneumoniae* isolates from companion animals (*n* = 105)

			Percentage of isolates with characteristic^a^				
AMR genes	Phenotype	Gene name	ESBL isolates (*n* = 52)	AmpC isolates^b^ (*n* = 34)	ESBL + AmpC isolates^b^ (*n* = 7)	OXA-48 isolates^b^ (*n* = 12)	Total (*n* = 105)
*bla*_CTX-M_	ESBL	*bla*_CTX-M-15_	80.8 (42)	0.0 (0)	85.7 (6)	16.7 (2)	47.6 (50)
		*bla*_CTX-M-14_	7.7 (4)	0.0 (0)	0.0 (0)	0.0 (0)	3.8 (4)
		*bla*_CTX-M-1_	5.8 (3)	0.0 (0)	0.0 (0)	0.0 (0)	2.9 (3)
		*bla*_CTX-M-3_	1.9 (1)	0.0 (0)	0.0 (0)	0.0 (0)	1.0 (1)
*bla*_SHV_	ESBL	*bla*_SHV-2_	1.9 (1)	0.0 (0)	0.0 (0)	0.0 (0)	1.0 (1)
		*bla*_SHV-12_	1.9 (1)	0.0 (0)	1.0 (1)	0.0 (0)	1.9 (2)
*bla*_OXA_		*bla*_OXA-10_	0.0 (0)	5.9 (2)	0.0 (0)	0.0 (0)	1.9 (2)
*bla*_DHA_	AmpC	*bla*_DHA-1_	0.0 (0)	91.2 (31)	85.7 (6)	75.0 (9)	44.8 (47)
*bla*_CMY_		*bla*_CMY-2_	0.0 (0)	8.8 (3)	14.3 (1)	0.0 (0)	3.8 (4)
*bla*_OXA_	Carbapenemase	*bla*_OXA-48_	0.0 (0)	0.0 (0)	0.0 (0)	100.0 (12)	11.4 (12)

a Numbers in parentheses are the actual number of isolates.

b Certain isolates may carry several resistance genes, so that the total number is not always equal to the sum of the numbers in the different cells for each column.

**Table 2.** Antimicrobial resistance proportions among *K. pneumoniae* isolates

		Percentage of resistant isolates^a^				
Antimicrobial class	Antimicrobial	ESBL isolates (*n* = 52)	AmpC isolates (*n* = 34)	ESBL + AmpC isolates (*n* = 7)	OXA-48 isolates (*n* = 12)	All isolates (*n* = 105)
Penicillin	Amoxicillin	49.5 (52)	32.4 (34)	6.7 (7)	11.4 (12)	100.0 (105)
Penicillin	Piperacillin	49.5 (52)	31.4 (33)	6.7 (7)	11.4 (12)	99.0 (104)
Penicillin	Ticarcillin	49.5 (52)	32.4 (34)	6.7 (7)	11.4 (12)	100 (105)
Penicillin + inhibitor	Amoxicillin + clavulanic acid	37.1 (39)	32.4 (34)	6.7 (7)	11.4 (12)	87.6 (92)
Penicillin + inhibitor	Piperacillin + tazobactam	1.0 (1)	6.7 (7)	1.0 (1)	11.4 (12)	20 (21)
Penicillin + inhibitor	Ticarcillin + clavulanic acid	45.7 (48)	30.5 (32)	6.7 (7)	11.4 (12)	95.2 (100)
1GC	Cefalotin	49.5 (52)	30.5 (32)	6.7 (7)	11.4 (12)	100 (105)
2GC	Cefuroxime	48.6 (51)	31.4 (33)	6.7 (7)	10.5 (11)	97.1 (102)
2GC	Cefoxitin	5.7 (6)	31.4 (33)	5.7 (6)	9.5 (10)	52.4 (55)
3GC	Cefotaxime	42.9 (45)	7.6 (8)	5.7 (6)	2.9 (3)	59.0 (62)
3GC	Ceftiofur	49.5 (52)	21.0 (22)	6.7 (7)	16.2 (17)	87.6 (92)
3GC	Ceftazidime	39.0 (41)	29.5 (31)	6.7 (7)	9.5 (10)	84.8 (89)
4GC	Cefepime	46.7 (49)	1.0 (1)	6.7 (7)	1.9 (2)	56.2 (59)
4GC	Cefquinome	46.7 (49)	1.0 (1)	5.7 (6)	6.7 (7)	60.0 (63)
Carbapenem	Ertapenem	0 (0)	11.4 (12)	0 (0)	10.5 (11)	21.9 (23)
Monobactam	Aztreonam	45.7 (48)	26.7 (28)	6.7 (7)	6.7 (7)	85.7 (90)
Aminoglycosides	Streptomycin	38.1 (40)	10.5 (11)	3.8 (4)	1.0 (1)	53.3 (56)
Aminoglycosides	Kanamycin	29.5 (31)	15.2 (16)	4.8 (5)	6.7 (7)	56.2 (59)
Aminoglycosides	Amikacin	0 (0)	0 (0)	1.9 (2)	0 (0)	1.9 (2)
Aminoglycosides	Apramycin	0 (0)	10.5 (11)	0 (0)	8.6 (9)	19.0 (20)
Aminoglycosides	Gentamicin	31.4 (33)	6.7 (7)	4.8 (5)	1.9 (2)	44.8 (47)
Aminoglycosides	Tobramycin	41.9 (44)	22.9 (24)	4.8 (5)	9.5 (10)	79.0 (83)
Aminoglycosides	Netilmicin	1.9 (2)	1.0 (1)	1.9 (2)	0 (0)	5.7 (6)
Amphenicol	Chloramphenicol	15.2 (16)	27.6 (29)	1.9 (2)	8.6 (9)	53.3 (56)
Amphenicol	Florfenicol	9.5 (10)	25.7 (27)	2.9 (3)	8.6 (9)	45.7 (48)
Tetracyclines	Tetracycline	34.3 (36)	23.8 (25)	5.7 (6)	2.9 (3)	66.7 (70)
Folic acid inhibitors	Sulfonamides	45.7 (48)	31.4 (33)	6.7 (7)	10.5 (11)	94.3 (99)
Folic acid inhibitors	Trimethoprim	43.8 (46)	12.4 (13)	6.7 (7)	1.9 (2)	64.8 (68)
Quinolones	Nalidixic acid	42.9 (45)	27.6 (29)	6.7 (7)	10.5 (11)	87.6 (92)
Fluoroquinolones	Enrofloxacin	39.0 (41)	25.7 (27)	6.7 (7)	9.5 (10)	81.0 (85)
Fluoroquinolones	Ofloxacin	42.9 (45)	27.6 (29)	6.7 (7)	10.5 (11)	87.6 (92)

1GC, first-generation cephalosporins; 2GC, second-generation cephalosporins; 3GC, third-generation cephalosporins; 4GC, fourth-generation cephalosporins.

a Numbers in parentheses are the actual number of isolates.

### Virulence determinants

The complete sequence of the yersiniabactin siderophore synthesis gene cluster *ybt-fyu-irp* was detected in 62.9% (*n* = 66) of isolates (Table S1 and S3), while the ferric uptake operon system (*kfuABC*) was found in 29.5% (*n* = 31) of isolates (Table S3). Two (1.9%) isolates recovered from horses and assigned to ST127-KL30/CMY-2 carried virulence genes coding for yersiniabactin (*ybt1*7), colibactin (*clb*3), the allantoinase cluster (*allABCDRS*) and the biofilm formation type 3 fimbriae gene cluster (*mrkABCD*). Only one ESBL/*mcr-9*-positive ST268-KL20 isolate, recovered from a horse, was found to be hypervirulent, containing virulence genes coding for the regulator of mucoid phenotype gene *rmpA*, yersiniabactin (*ybt* unknown), colibactin (*clb3*), aerobactin (*iucABCD*) and salmochelin operon (*iroBCDN*).

### Population structure and phylogeny

**Figure 1.**
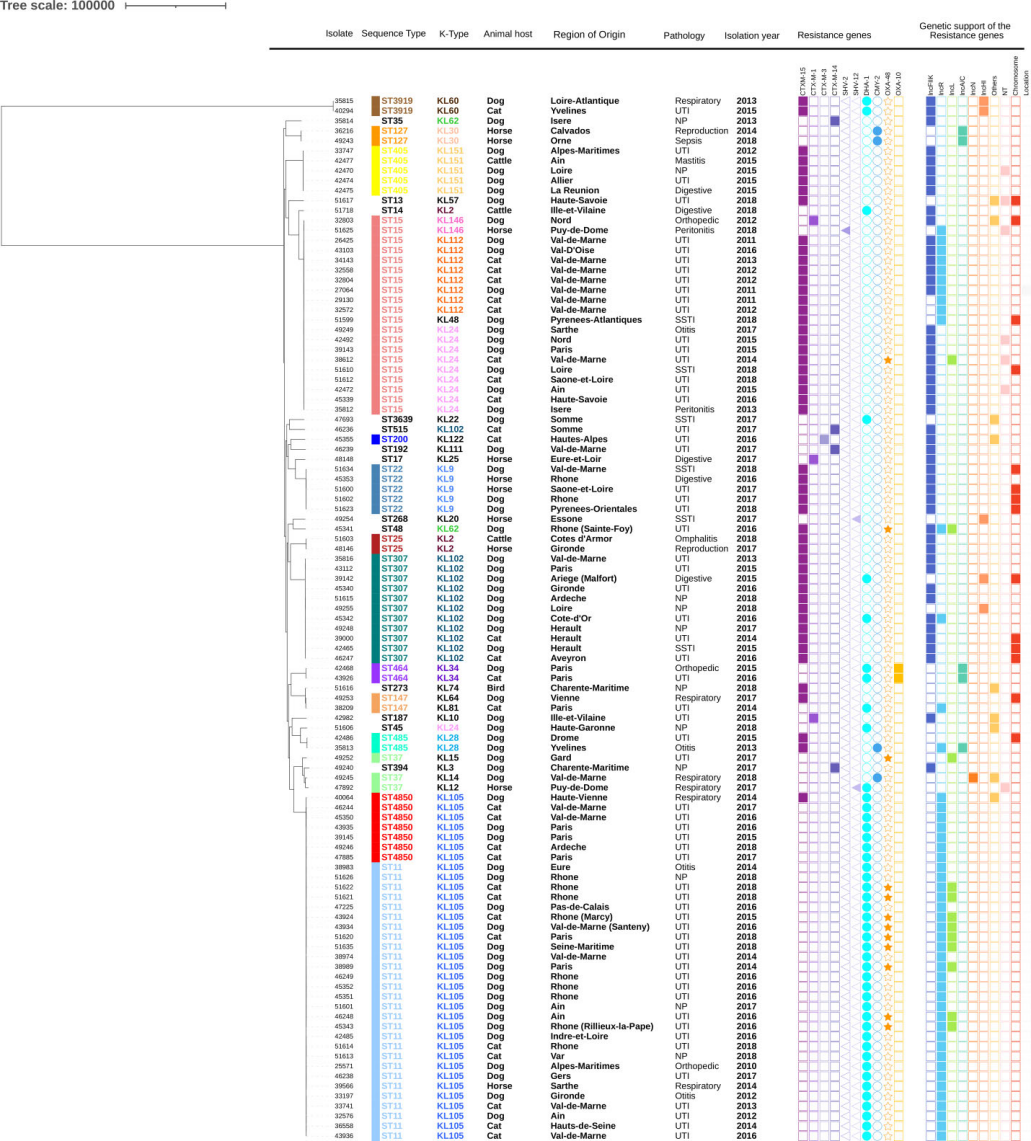
Maximum-likelihood phylogeny of *K. pneumoniae* isolates from companion animals. The phylogenetic tree was constructed based on nucleotide sequence alignments of 4214 core genes. The tree was rooted using a *K. oxytoca* isolate from our collection that was later removed. Branch lengths represent the number of nucleotide substitutions per site (scale, 100 000 substitutions per site). The two KL60 isolates are *Klebsiella variicola* subsp. *variicola*. Metadata columns include sequence types shown with coloured bars (each colour correspond to one ST), geographical origin of isolates, information on the pathology of the animals and the isolation year. ESBL/AmpC/carbapenemase genes as well as plasmid content are indicated with rectangles, triangles, circles or stars denoting the presence (filled shapes) or absence (empty shapes) of the gene. This figure appears in colour in the online version of *JAC* and in black and white in the print version of *JAC*.

The genetic diversity revealed 27 different STs (Figure 1). The most frequent STs were ST11 and its SLV ST4850 (33.3%, *n* = 35), followed by ST15 (19.0%, *n* = 20) and ST307 (10.5%, *n* = 11). Other, minor STs were also identified, such as ST22 (4.8%, *n* = 5), ST405 (4.8%, *n* = 5) and ST37 (2.9%, *n* = 3), and 20 isolates for which the ST was detected in only one or two isolates (Figure 1 and Table S1). Isolates harbouring *bla*_CTX-M-15_ were predominantly assigned to ST15 (*n* = 18), ST307 (*n* = 11), ST405 (*n* = 5) and ST22 (*n* = 5), whereas isolates carrying *bla*_DHA-1_ principally belonged to ST11 (*n* = 28), ST4850 (*n* = 7) and ST464 (*n* = 2). The *bla*_OXA-48_ was associated with ST11 (*n* = 9), ST15 (*n* = 1) or ST48 (*n* = 1), when co-harboured with *bla*_DHA-1_ and *bla*_CTX-M-15_ respectively, and ST37 (*n* = 1) when carried alone (Figure 1).

A total of 28 distinct K-types were identified, including three KL2 (2.9%), a K-type typically present in hypervirulent *K. pneumoniae* clones. All ST11 and ST4850 isolates were KL105 and all ST307 isolates were KL102, while ST15 isolates principally presented K24 (*n* = 9) and KL112 (*n* = 8).

The maximum-likelihood phylogenetic tree based on the recombination-free alignment of 4214 core genes showed three large clusters corresponding to ST11/ST4850, ST15 and ST307 (Figure 1). Within each cluster, ST11/ST4850 isolates differed by a maximum of 325 SNPs, ST15 isolates by a maximum of 232 SNPs and ST307 only 86 SNPs. Smaller clusters corresponding to other STs, such as ST405 or ST22, and 12 singletons not clustering into any major ST-associated group were also observed. The phylogenetic tree (Figure 1) as well as the MST (Figure S1) revealed that the clustering of isolates was independent of the year of isolation, the animal source or infection type.

### Cross-sectoral phylogenetic comparisons of ST11, ST15 and ST307 K. pneumoniae

**Figure 2.**
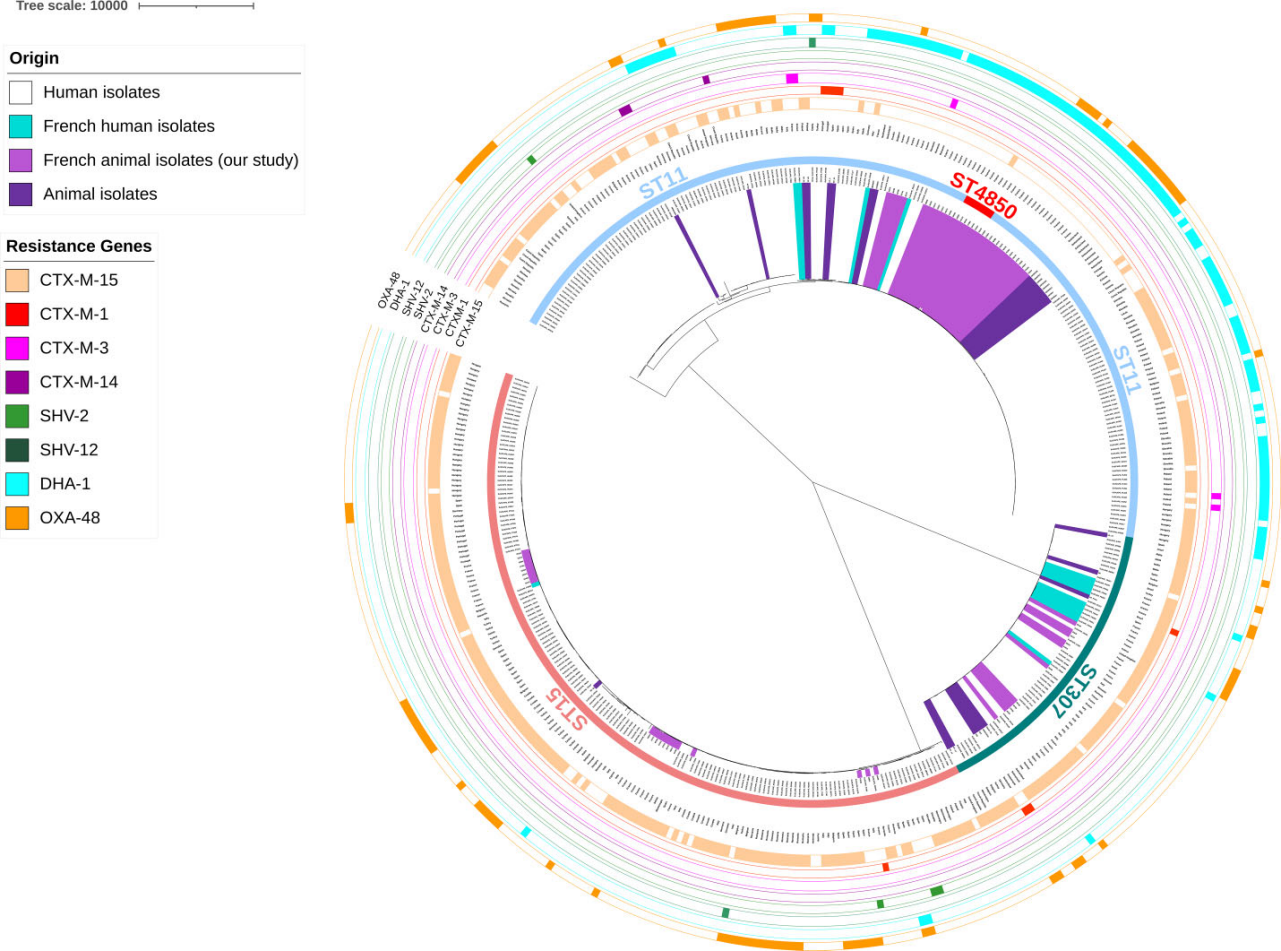
Phylogenetic tree of the ST15, ST11 and ST307 *K. pneumoniae* isolates from companion animals placed in the context of a global human collection from the EuSCAPE) study^25^ as well as other animal isolates belonging to the same STs.^26^ The phylogenetic tree was constructed based on nucleotide sequence alignments of 4254 core genes. Sequence types are shown with coloured bars, each colour corresponds to one ST. ESBL/AmpC/carbapenemase genes are indicated with coloured bars denoting the presence (filled bars) or absence (empty bars) of the gene. The source of the isolates is indicated by coloured shading. This figure appears in colour in the online version of *JAC* and in black and white in the print version of *JAC*.

The 66 ST11/ST4850, ST15 and ST307 *K. pneumoniae* genomes of our collection were compared with published human (*n* = 326) and animal (*n* = 15) *K. pneumoniae* genomes of the same STs. The clonal relatedness was evaluated using a maximum-likelihood phylogenetic tree on the recombination-free alignment of 4254 core genes. The phylogenetic tree showed that human and animal *K. pneumoniae* isolates grouped in three large clusters according to their ST (Figure 2). ST11/ST4850 isolates differed by a maximum of 4633 SNPs, ST15 isolates by a maximum of 833 SNPs and ST307 only 166 SNPs. The high number of SNPs observed in ST11 is largely due to two EuSCAPE isolates (one from Czech Republic and the second one from Romania), and these divergences were not due to sequencing problems. Among animal isolates, a large cluster of 31 ST11 isolates from France and Switzerland was observed, which differed by only 1–46 SNPs. The SNP differences between animal isolates were generally smaller than those between animal and human isolates. None of the French animal isolates shared less than 21 SNPs with any of the human isolates of the EuSCAPE study. Nevertheless, several animal isolates were closely related to human ones. For example, the only human French ST15 isolate identified in the EuSCAPE collection (FR002) clustered with eight French animal isolates, from five cats and three dogs (26425, 27064, 29130, 32572, 32804, 34143, 32558 and 43103), and differed from them by 30–72 SNPs. Among ST11, five French animal isolates, from three cats and two dogs (32576, 36558, 33741, 33197 and 43936) and one French human isolate (FR049) showed 21–52 SNPs differences. Among ST307, one French human isolate (FR030) and one French animal isolate from a dog (45340) only differed by 25 SNPs.

The MST showed a clustering by country and by host, especially for ST11/ST4850, which presented as an animal sub-lineage in which the French and non-French animal genomes mostly grouped together (Figure S2). The MST also confirmed the rare clustering of French animal and human isolates. Such clustering was found twice in ST15 (the first cluster including five animal isolates – 26425, 27064, 29130, 32572 and 32804, from three cats and two dogs – and the FR002 isolate, the second cluster including the 49249 animal isolate from a dog and the human ES219 isolate from Spain) and once in ST307 [including six animal isolates from France (two cats and two dogs) and the Czech Republic (two dogs) – 39000, 42465, 46247, 49248, KP_13, KP_14 – and two human isolates from Spain – EuSCAPE_ES148, EuSCAPE_ES149 – presenting 0–3 SNP differences].

### Plasmid characterization

*K. pneumoniae* isolates harboured one to four plasmids whose sizes ranged from 45 to 350 kb (Table S1 and Table S4). In ST15, *bla*_CTX-M-15_ was carried by 45–70 kb IncR (*n* = 3/20) or 100–250 kb IncFIIk plasmids (*n* = 9/20) or both plasmids in the same cell (*n* = 6/20). *bla*_CTX-M-15_ was carried by 100–250 kb IncFIIk plasmids in ST307 (*n* = 9/11), ST405 (*n* = 5/5) and ST22 (*n* = 5/5), or sporadically by 250 kb IncHI1 plasmids or 50 kb IncI1 plasmids in isolates from different STs. The *bla*_DHA-1_ gene was mostly identified on 45–70 kb IncR plasmids in all ST11 and all ST4850 isolates, or a 180 kb IncA/C plasmid in the two ST464 isolates. Finally, the *bla*_OXA-48_ gene was always located on ∼60 kb IncL plasmids.

## Discussion

Reports on ESBL-, AmpC- and/or carbapenemase-producing *K. pneumoniae* from companion animals are limited, and mainly restricted to case reports of human high-risk clones, suggesting human-to-animal spillovers. We present the largest collection of ESBL-, AmpC- and/or carbapenemase-producing *K. pneumoniae* from companion animals, with a prominent result that 59 out of 105 (56.2%) isolates belonged to the human-associated MDR ST11, ST15 and ST307 lineages. Our results provide further evidence that ST307, which is now widely reported in humans after having gone unnoticed for 20 years,^17^ is also widely distributed among companion animals.^1,16–19^ Other minor clones not previously described in animals were also detected, demonstrating the circulation of various *K. pneumoniae* lineages contributing to the spread of ESBL, AmpC and/or carbapenemase genes in pets. Most of them have also been described in humans, suggesting that numerous human-associated clones also have the capacity to infect the animal host. The notable exception is ST4850, but it is a rare ST that evolved from an ST11 ancestor by a single point mutation of the *rpoB* locus. The dissemination routes between humans and animals still remain to be studied for both pathogenic and commensal isolates, since the sharing of strains can also happen between healthy individuals in a household.^2^

The *bla*_CTX-M-15_ gene was the dominant β-lactamase gene, accounting for 80.8% of ESBL-producing *K. pneumoniae* isolates. This is the most commonly reported gene worldwide from ESBL-positive *K. pneumoniae* clinical isolates,^1,30,31^ and such a high prevalence in companion animals consolidates sporadic findings in Italy,^10^ Portugal,^1^ Germany,^8^ France,^7^ Spain,^21^ Switzerland,^32^ Brazil,^33^ Japan,^16^ and South Korea.^19^ All ST307 (*n* = 11/11) and all but two ST15 (*n* = 18/20) presented the *bla*_CTX-M-15_ gene, as observed previously.^1,7–13,16,18,19,34^.Almost all AmpC-type β-lactamase genes were *bla*_DHA-1_ (91.2%) and belonged to ST11 or ST4850, in line with previous observations,^9,14,15,34^ while *bla*_CMY-2_ was sporadically found (8.8%) in diverse STs, among which was ST37, also previously found in a dog in Portugal.^34^ We report 12 *bla*_OXA-48_-producing *K. pneumoniae* in companion animals, which adds to those previously detected ones in Germany in 2012,^9,35^ and Spain.^21^ The *bla*_OXA-48_ gene was predominantly found in ST11 isolates (*n* = 9) co-harbouring *bla*_DHA-1_ but only once in an ST15 isolate, which contrasts with findings in Germany, where *bla*_DHA-1_-positive ST11 and *bla*_CTX-M-15_-positive ST15 accounted for respectively 19.8% and 75.6% of the *bla*_OXA-48_-producing isolates.^9,20^

Multiple co-resistances to non-β-lactam antimicrobials were also found, such as to sulphonamides or fluoroquinolones. Interestingly, one ST37 isolate retrieved from a horse presented the *armA* gene, and harboured *bla*_DHA-1_ and *bla*_SHV-12_ on a non-typeable plasmid of ∼250 kb. Plasmid-encoded 16S rRNA methyltransferases, conferring resistance to practically all aminoglycosides, are still very rare in *K. pneumoniae* from animal origin.^36^ Until now, they have only been identified in Spain in ST11 isolates from cats and dogs,^14^ in China in two isolates recovered from a dog and a cat^36^ and in Japan in ST15 and ST37 isolates (also harbouring the *bla*_DHA-1_ and *bla*_SHV-12_ genes) from companion animals.^11,37,38^ Finally, colistin resistance was also observed in one isolate, despite the absence of colistin use in French companion animals, and was attributed to the disruption of the *mgrB* gene. The presence of the *mcr-9* gene was also found in one isolate, but this gene did not confer colistin resistance as expected based on previous reports.

Historically, MDR and virulent isolates have represented non-overlapping populations of *K. pneumoniae*, with sporadic reports of convergent hypervirulent and MDR strains.^39^ However, the detection of isolates that are both highly pathogenic and resistant to most available antibiotics has increased recently.^40,41^ Here, one hypervirulent ESBL-positive ST268 isolate as well as two virulent AmpC-positive ST127 isolates were identified. This provides evidence that convergence of hypervirulence and AMR has to be surveyed also in animals.

In line with previous studies, *bla*_CTX-M-15_, *bla*_DHA-1_ and *bla*_OXA-48_ were strongly associated with IncFIIk,^10^ IncR^14,15^ and IncL^9,20^ plasmids, respectively. A few *bla*_CTX-M-15_ genes were also located on IncR plasmids, an association already found in isolates from France and Italy.^7,10,42^ Interestingly, eight isolates carried two copies of *bla*_CTXM-15_ (on IncFIIK and IncR plasmids), and ten additional isolates presented one plasmidic and one chromosomally integrated copy of *bla*_CTX-M-15_. Extra copies of resistance genes have been connected to a higher resistance level or bacterial response towards increased antibiotic concentration.^43^ We also detected three isolates that only had *bla*_CTX-M-15_ located in the chromosome. Descriptions of ESBL genes located in the chromosome of *K. pneumoniae* are still very rare compared with *E. coli*, even though such an integration has already been reported in Spain, in Germany and in the USA.^44–46^ IncR plasmids carrying the *bla*_DHA-1_ gene were found in all isolates from ST11 and its derived ST4850. This association has been detected in companion animals in Spain,^14^ Italy,^10^ and Switzerland^15^ and in clinical isolates in France.^47,48^ Studies on human isolates in France determined that more than half of the *bla*_DHA-1_-encoding strains harboured an IncR plasmid and belonged to ST11, similarly to our study. Besides, several studies showed that IncR plasmids have disseminated especially well in *K. pneumoniae* isolates belonging to ST11 and ST258.^49^ Interestingly, isolates co-harbouring *bla*_CTX-M-15_ and *bla*_DHA-1_ genes were assigned to other STs than ST11 or ST15. Finally, the *bla*_OXA-48_ gene was always located on IncL plasmids of ∼60 kb, which is consistent with the literature.^50^

The first maximum-likelihood phylogenetic tree grouped animal isolates into three main clusters (ST11/ST4850, ST15 and ST307), which have also been the most frequently detected ones in pets until now. Even though this clustering was largely independent of the year of isolation, animal host or infection, two geographical subclusters were identified, possibly indicating a clonal spread in specific areas: the first one included eight ST15 isolates collected in the Val de Marne between 2011 and 2014 with a low number of different cgMLST alleles (ranging from 1 to 17) and the second one included nine ST11 isolates collected in the Rhône administrative Department between 2015 and 2018, these also showed a low number of SNP differences by cgMLST, ranging from 1 to 25. These data most likely do not highlight direct transfers of ESBL/AmpC/carbapenemase-producing *K. pneumoniae* clones among companion animals with unrelated epidemiological histories but may rather reflect indirect and unclarified transmissions.

Animal *K. pneumoniae* genomes belonging to the three most prevalent STs in our study and *K. pneumoniae* genomes of humans and animals of varied geographical origins were analysed to explore animal/human transmission dynamics. Four groups of very closely related French human and animal isolates belonging to the three STs were detected: these isolates displayed ≤72 SNP differences within the ST15 cluster, ≤52 within the ST11 cluster, and ≤25 or ≤105 SNP differences in the two subclusters within ST307. David *et al*.^25^ suggested that <21 SNPs indicates a hospital outbreak; our data might thus not indicate direct human-to-animal transmission, but the circulation of genetically related clones at a larger scale. However, a much larger set of data would be needed to clarify transmission. Interestingly enough, animal isolates were principally not intermingled across the whole phylogeny within each ST but rather grouped into small subclusters, which may indicate different transmission chains of *K. pneumoniae* within animal populations, with the caveat that undersampling of humans in contact, i.e. pet owners or veterinarians, may mask cross-transmissions. Notably, a major subcluster of ST11/ST4850 grouped animal isolates from this and other studies, possibly reflecting an ST11 sub-population particularly well-adapted to the animal host or which benefited from particular transmission opportunities. Of note, all animal ST11 presented the KL105 capsule locus structure, while only 66/139 (47.5%) of the human isolates carried it, reinforcing the hypothesis of a homogeneous sub-lineage particularly adapted to the animal host, as already pointed out by Brilhante *et al*.^26^ ST15 and ST307 animal isolates appeared more scattered, which may either indicate that these STs are less adapted to animal hosts compared with ST11, or simply reflect the still too limited number of genomes available from animals for relevant genomic comparisons.

In conclusion, our data demonstrate the dissemination of *bla*_CTX-M-15_-carrying ST15 and ST307, and *bla*_DHA-1_-carrying ST11 *K. pneumoniae* clones in animal populations. A phylogenomic analysis suggests that the occurrence of these lineages is not a simple spillover from the human reservoir. Indeed, a specific subcluster of ST11/KL105 that encompasses among others the OXA-48-producing *K. pneumoniae*, might well be animal-adapted. The presence of OXA-48 in pets in the absence of carbapenem use, and the emergence of (hyper)virulent ESBL/AmpC *K. pneumoniae* mirroring the emergence of ‘dual risk’ *K. pneumoniae* strains in humans, is of concern. Our study underlines the need for further large-scale cross-sectoral genomic studies on *K. pneumoniae* to better understand transmission within and between human and animal populations.

## Supplementary Material

dkac041_Supplementary_Data

## Supplementary data

Figures S1 and S2 and Tables S1 to S4 are available as Supplementary data at *JAC* Online.
