# Supplementary material for: Comparative phylogenomics of ESBL-, AmpC- and carbapenemase-producing Klebsiella pneumoniae originating from companion animals and humans
Source: J Antimicrob Chemother. 2022 Feb 28;77(5):1263–71. doi: 10.1093/jac/dkac041 (PMC9047677; doi:10.1093/jac/dkac041)

**Supplementary data**

Tables S1 to S4 are available in the separate Excel file.

**TABLE S1.** Summary of associated metadata and phenotypic and genotypic characteristics of ESBL/AmpC-producing and carbapenem resistant *K. pneumoniae* isolates from companion animals.

**TABLE S2.** Antimicrobial resistance gene distribution among ESBLs, AmpC and OXA-48 *K. pneumoniae isolates*.

**TABLE S3.** Distribution of virulence determinants and heavy metal tolerance genes among ESBL/AmpC- and carbapenem-resistant *K. pneumoniae* isolates from companion animals.

**TABLE S4.** Overview of plasmid families associated with ESBLs, AmpC and OXA-48 in *K. pneumoniae* isolates from companion animals.

**FIGURE S1.** Minimum spanning tree (MST) of *K. pneumoniae* isolates from companion animals was calculated based on the cgMLST scheme available on BIGSdb [^29^](#_ENREF_29). Each node whose size is proportional to a number of isolates, corresponds to a single sequence type profile based on 629 core genes. Nodes are colour-coded according to sequence types defined by MLST as described in the legend. All nodes are linked (black lines) sequentially to the most closely related node with the highest number of cgMLST alleles in common. The numbers along edges correspond to the number of different alleles in the cgMLST.


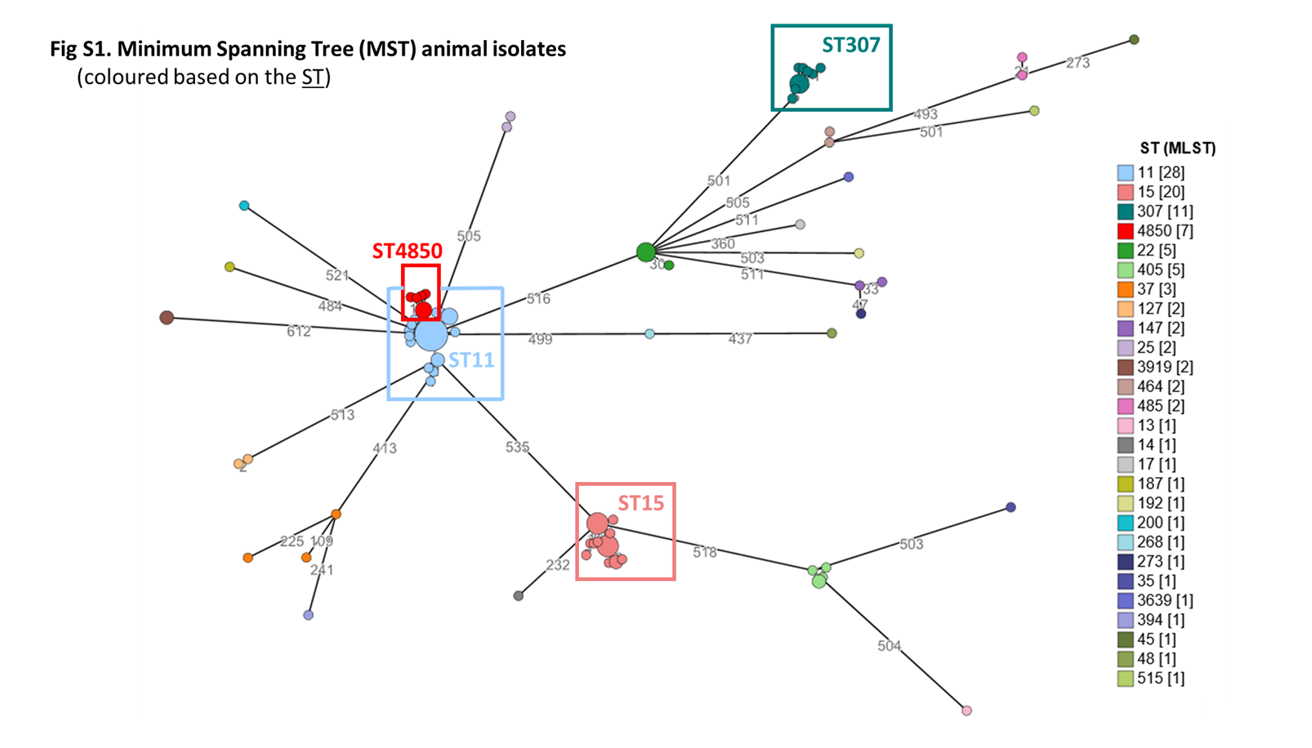
**FIGURE S2.** Minimum spanning tree (MST) of ST15, ST11 and ST307 *K. pneumoniae* isolates from companion animals placed in the context of a global human collection from the EuSCAPE study [^25^](#_ENREF_25) as well as other animal isolates belonging to the same STs [^26^](#_ENREF_26) based on cgMlST scheme which consists of 629 loci available in BIGSdb (a) colour-coded by STs according to the legend (b) colour-coded by country of isolation according to the legend.


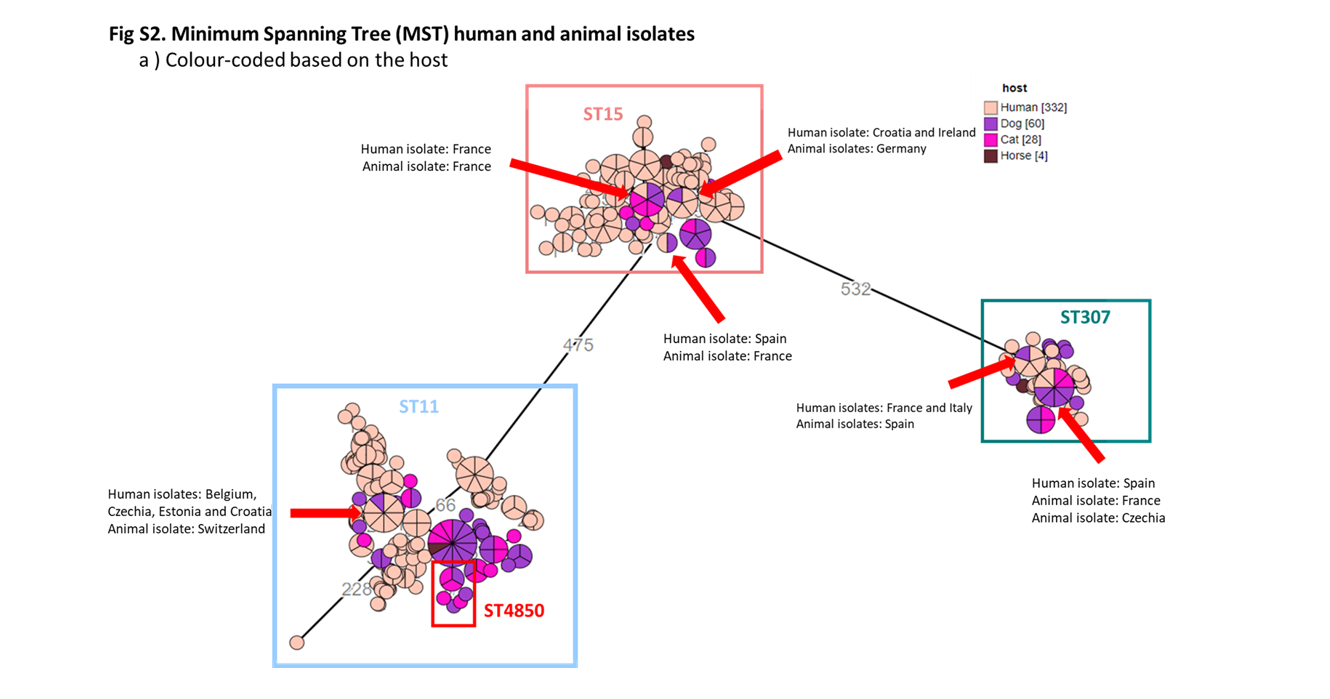


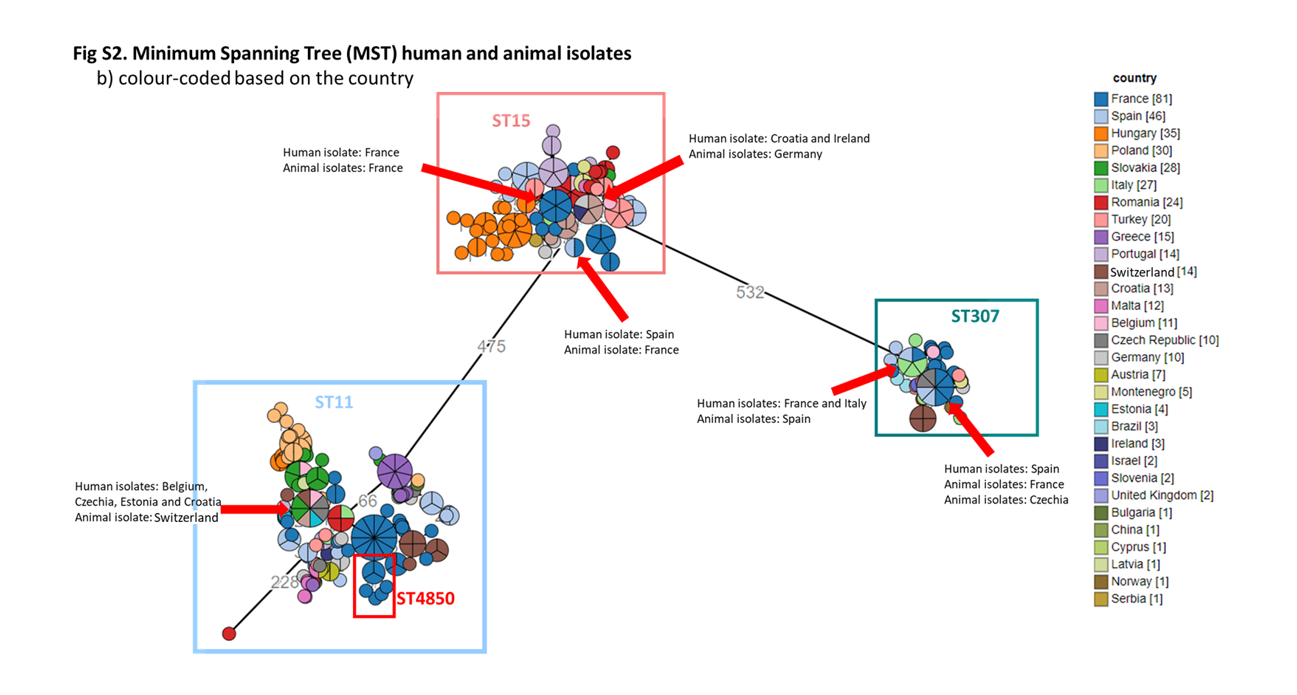

Supplement: dkac041_Supplementary_Data [file dkac041_supplementary_data.zip › 21-1070-Suppl-data-1.docx]
